# Integrative QTL mapping and transcriptomic profiling uncover molecular mechanisms underlying lodging resistance in *Brassica napus*

**DOI:** 10.1093/hr/uhag201

**Published:** 2026-05-20

**Authors:** Mingli Wu, Huaixin Li, Liyun Miao, Yutian Xia, Xinmin Liu, Xin Cheng, Jianshuo Li, Shipeng Fan, Dongqing Zhang, Xiaoling Dun, Maoteng Li

**Affiliations:** Institute of Biological Seed Industry, Xianghu Laboratory, Hangzhou 311231, China; Key Laboratory of Molecular Biophysics of the Ministry of Education, College of Life Science and Technology, Huazhong University of Science and Technology, Wuhan 430074, China; Institute of Biological Seed Industry, Xianghu Laboratory, Hangzhou 311231, China; Oil Crops Research Institute, Chinese Academy of Agricultural Sciences, Wuhan 430062, China; Key Laboratory of Molecular Biophysics of the Ministry of Education, College of Life Science and Technology, Huazhong University of Science and Technology, Wuhan 430074, China; Key Laboratory of Molecular Biophysics of the Ministry of Education, College of Life Science and Technology, Huazhong University of Science and Technology, Wuhan 430074, China; Key Laboratory of Molecular Biophysics of the Ministry of Education, College of Life Science and Technology, Huazhong University of Science and Technology, Wuhan 430074, China; Key Laboratory of Molecular Biophysics of the Ministry of Education, College of Life Science and Technology, Huazhong University of Science and Technology, Wuhan 430074, China; Key Laboratory of Molecular Biophysics of the Ministry of Education, College of Life Science and Technology, Huazhong University of Science and Technology, Wuhan 430074, China; Key Laboratory of Molecular Biophysics of the Ministry of Education, College of Life Science and Technology, Huazhong University of Science and Technology, Wuhan 430074, China; Institute of Biological Seed Industry, Xianghu Laboratory, Hangzhou 311231, China; Oil Crops Research Institute, Chinese Academy of Agricultural Sciences, Wuhan 430062, China; Institute of Biological Seed Industry, Xianghu Laboratory, Hangzhou 311231, China; Key Laboratory of Molecular Biophysics of the Ministry of Education, College of Life Science and Technology, Huazhong University of Science and Technology, Wuhan 430074, China

## Abstract

Lodging remains a significant agronomic challenge that limits the yield potential of *Brassica napus* globally, making the elucidation of its molecular basis critical for breeding new germplasm. Lignocellulose components (including lignin, cellulose, and hemicellulose) play an essential role in the mechanical strength of the stem, and they are regarded as the key determinants of lodging. In a doubled haploid (DH) population, 61 consensus quantitative trait loci (QTLs) associated with the lignocellulose content in the stem were identified through QTL analysis. Integrating QTL mapping with transcriptomic data revealed 1035 candidate genes potentially involved in stem lignocellulose biosynthesis, of which 52 high-confidence candidates were selected. Among them, *BnaC01.MYB85*, *BnaA04.WRKY12*, and *BnaC03.ASMT* exhibited stem-specific expression. A stem-specific lignocellulose biosynthetic pathway characterized by the coordinated expression of these high-confidence genes was delineated. Haplotype analysis in a natural population showed that *BnaC03.ASMT* segregated into two major haplotypes, with hap01 exhibiting a significantly higher hemicellulose content (HC) than hap02. These findings provide novel insights into the genetic regulation of lodging resistance and offer a valuable foundation for breeding *B. napus* cultivars with higher stem strength and lodging tolerance.

## Introduction

Rapeseed (*Brassica napus*, A_n_A_n_C_n_C_n_) is one of the most vital oil-bearing crops in the world, and it ranks second in global cultivation area among oilseed crops, surpassed only by soybean. Renowned for its substantial economic value, rapeseed serves as a primary source of high-quality edible vegetable oil [1]. However, lodging has emerged as a major constraint in rapeseed production, especially with the increasing occurrence of extreme weather events, severely compromising both the yield and mechanical harvesting efficiency [2]. Previous studies have reported that lodging reduces rapeseed yield by 10%–30%, with a comparable decrease in oil content [3]. In addition to its agricultural significance, *B. napus* is cultivated as an ornamental plant. Lodging can reduce its ornamental value by disrupting the plant architecture and diminishing the visual uniformity of flowering landscapes.

Lodging is generally categorized as stem lodging and root lodging. Previous studies have indicated that stem lodging poses a greater threat to crop productivity than root lodging [4–6]. Stem strength, particularly that of the basal internodes, is considered a key determinant of lodging resistance [7]. Recently, quantitative trait locis (QTLs) associated with stem strength have been investigated, and several causal genes have been identified. In wheat, QTL mapping in a doubled haploid (DH) population revealed overlapping genomic *regions* controlling multiple stem biomechanical traits [8]. In soybean, nine QTLs associated with stem diameter were identified in 207 chromosome segment substitution lines (CSSLs), and the causal gene *Glyma.04G004100* was characterized using an integrated analysis of whole-genome resequencing, RNA sequencing (RNA-Seq), and *q*PCR [9]. In rice, a genome-wide association study (GWAS) of 550 accessions identified 89 QTLs controlling nine stem strength and leaf type-associated traits, and haplotype and functional annotation analyses revealed 21 candidate genes [10]. Similarly, in rapeseed, a GWAS identified 67 significantly associated QTLs and 71 candidate genes related to stem lodging traits, including stem breaking resistance, stem diameter, stem strength, and the lodging coefficient [11].

The mechanical strength of the basal stem is primarily determined by the composition and accumulation of structural carbohydrates, including soluble sugars, starch, cellulose, and lignin [12–14]. Lignin and cellulose have been widely reported to enhance the stem strength and lodging resistance in cereal crops, such as wheat, maize, and rice [15–18]. As a fundamental component of secondary cell walls, lignocellulose, which consists of lignin, cellulose, and hemicellulose, plays a critical role in mechanical support in plants [19–21]. Previous studies have conducted QTL mapping for the lignin content (LC), cellulose content (CC), and hemicellulose content (HC) in *B. napus*, employing both linkage and association analyses. However, most studies have focused on lignocellulose accumulation in seed tissues [22–24], and investigations of the lignocellulose content in stems, particularly in relation to lodging resistance, remain limited. Four novel candidate genes involved in stem lignin biosynthesis were identified in rapeseed by integrating GWAS with transcriptome sequencing [25]. In addition, 67 additive QTLs were identified for seven stem-related traits: stem LC, CC, HC, the S/G monolignol ratio, stem breaking force, breaking strength, and *Sclerotinia sclerotiorum* resistance [26]. Four QTLs associated with LC in the stalk and taproot were also detected on ChrA05, ChrA09, and ChrC09 based on 125 natural rapeseed accessions [27].

Recent advances in next-generation sequencing technologies have accelerated the development of genomics, transcriptomics, proteomics, and metabolomics. Integrating these approaches has facilitated the identification of key candidate genes and the elucidation of the regulatory mechanisms underlying agronomic traits [28–30]. RNA-Seq has emerged as a powerful high-throughput technique for transcriptome analysis, enabling the comprehensive profiling of dynamic gene expression patterns. When integrated with QTL mapping, RNA-Seq effectively narrows down candidate gene regions and improves the accuracy of candidate gene identification [31–35]. In *B. napus*, an integrated approach of QTL mapping and RNA-Seq identified four promising candidate genes (*BnaA10G0085600ZS*, *BnaA10G0156900ZS*, *BnaC03G0441700ZS*, and *BnaC08G0410600ZS*) as key regulators of leaf size through cell division regulation [31]. Additionally, 17 candidate genes potentially involved in seed color on ChrA09 and ChrC03 in *B. napus* were identified through QTL mapping and differential expression and co-expression network analyses [32].

Despite previous research on lodging resistance in *B. napus*, the underlying molecular mechanisms remain poorly understood. Specifically, comprehensive studies integrating multiomics approaches to systematically identify lodging-associated genes and biochemical pathways are still limited. This study aimed to address this gap by combining QTL mapping and comparative transcriptomics to reveal the genetic regulation of lignocellulose accumulation in rapeseed stems, thereby providing a more complete understanding of the lodging resistance mechanism in *B. napus*.

## Results

### Phenotypic variation in stem lignocellulose components

Substantial phenotypic variations (PVs) in stem lignocellulose components were observed in the KN DH population grown across six environments (2013, 2014, 2015 in Wuhan (WH) and Dali (DL); Table S2). LC, CC, and HC exhibited normal or near-normal distributions with transgressive segregation (Fig. 1A–C), indicating that these traits are quantitatively inherited and suitable for QTL mapping. The estimated broad-sense heritability (*H*^2^) of LC, CC, and HC exceeded 0.99 under six environments, suggesting that these traits are predominantly controlled by genetic factors.

**Figure 1:**
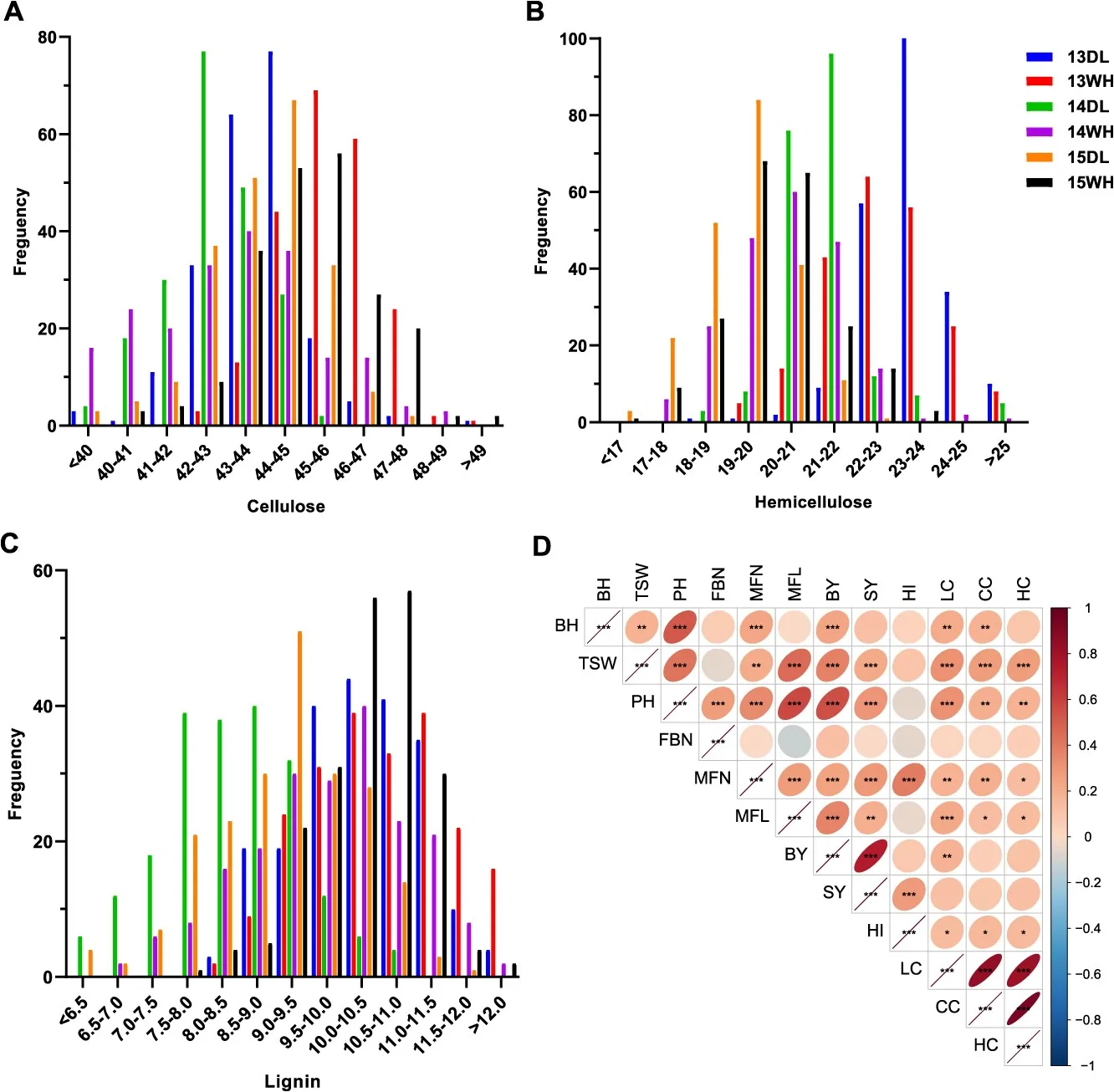
The frequency distribution of three lignocellulose components in multiple environments. (A–C) represent the distributions of CC (A), HC (B), and LC (C), respectively. The *x*-axis denotes phenotypic values, while the *y*-axis indicates the number of lines, with distinct environments differentiated by color-coded boxes. (D) represent the correlational analyses between three lignocellulose components and yield related traits. Abbreviations: BH, first branch height; TSW, thousand seed weight; PH, plant height; FBN, first effective branch number; MFN, main inflorescence silique number; MFL, main inflorescence effective length; BY, biomass yield per plant; SY, seed yield per plant; HI, harvest index. Statistical significance is indicated as follows: ^*^, *P* < 0.05; ^**^, *P* < 0.01; ^***^, *P* < 0.001.

Correlational analysis revealed significant positive associations among LC, CC, and HC (*r* > 0.82, *P* < 0.01), with the highest correlation observed between HC and CC (*r* = 0.93; Table S3). Correlations across environments were generally weaker than those observed within the same environment, reflecting the significant influence of environmental factors on lignocellulose components in rapeseed stems (Fig. S1). To explore the relationship between lignocellulosic composition and yield-related traits, data from WH collected over 3 years (2013–2015) were analyzed. All three lignocellulose components, particularly LC, showed positive correlations with most yield-related traits (Fig. 1D). These findings highlight the advantages of optimizing the lignocellulose content in *B. napus* stems; thus, enhancing lodging resistance could simultaneously improve yield performance.

### Genome-wide QTL mapping and integration of lignocellulose components

A genome-wide QTL analysis was performed for three stem lignocellulose components using phenotypic data collected over 3 years from WH and DL. Across six environments, 65 QTLs controlling LC (22), CC (22), and HC (21) were identified in 15 linkage groups, with the exception of ChrA07, ChrC04, ChrC08, and ChrC09 (Fig. 2, Table S4). The number of QTLs per linkage group ranged from 1 to 11, with the highest number detected on ChrA06 and ChrC01. The explained PV ranged from 3.80% to 30.06%, with the largest contribution from *qHC-14DL-2*, which exhibited a negative additive effect, indicating that the favorable allele was derived from the male parent Ken-C8. Meta-analysis condensed the 65 initial QTLs into 61 consensus QTLs, of which four were stable across environments, and the remaining 57 were environment-specific (Table S5).

**Figure 2:**
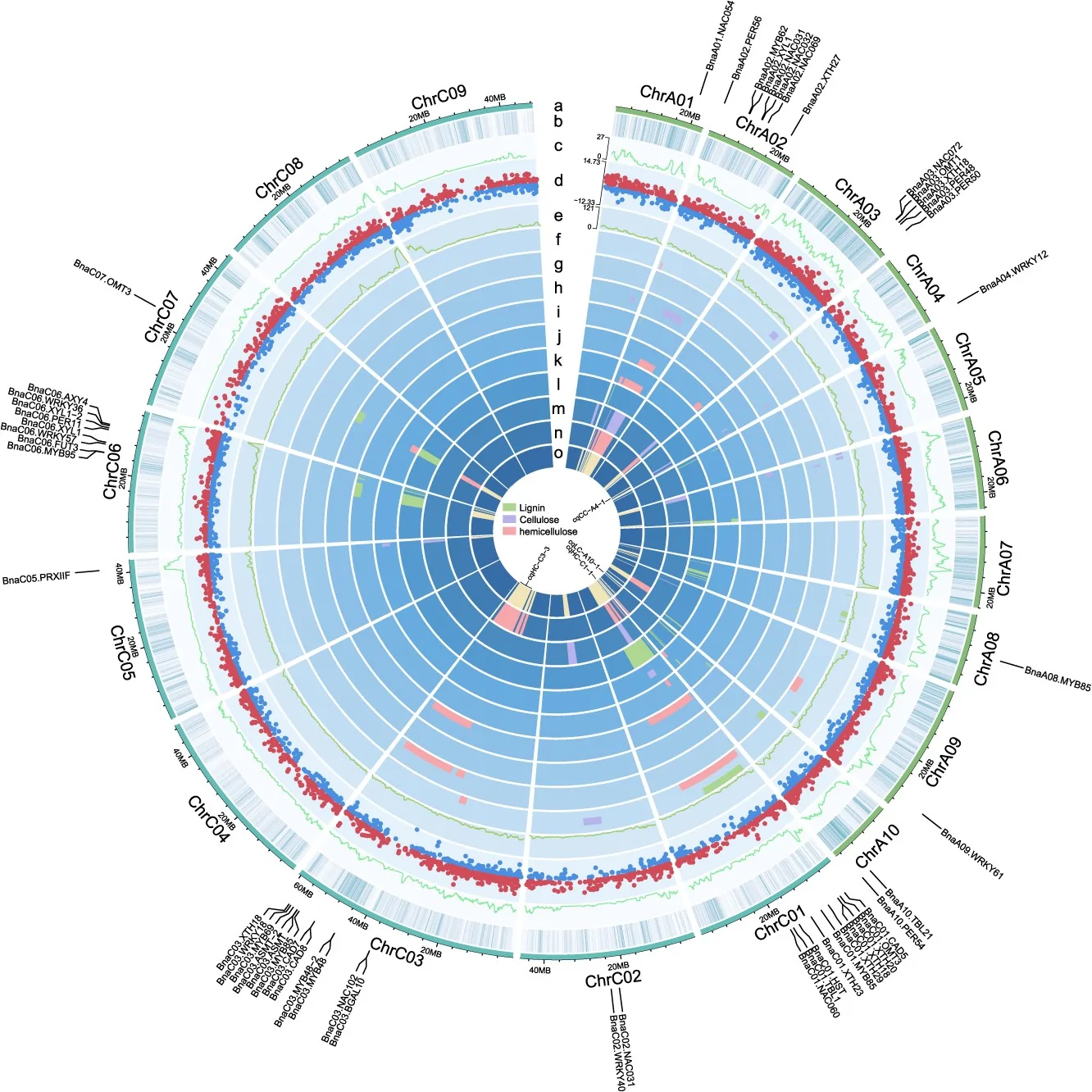
Integrative results of QTL mapping and transcriptomic sequencing data. The outermost circle displays the 52 high-confidence candidate genes. (A) 19 linkage groups; (B) Gene density; (C) Marker density; (D) The distribution of DEGs (Red and blue dots denote significantly up- and down-regulated genes, respectively.); (E) The density of DEGs; (F–K) The distribution of QTLs for LC, CC, and HC in different environments: (F) 13DL; (G) 13WH; (H) 14DL; (I) 14WH; (J) 15DL; (K) 15WH; and (L–N) consensus QTLs for LC (L), CC (M) and HC (N), respectively; (O) Unique QTLs. The innermost circle displays four environmental stability QTLs.

*cqHC-C1-1* was consistently detected in two environments (*qHC-13DL-5* and *qHC-14WH-1*), with PVs of 7.38% and 8.76%, respectively. Similarly, *cqHC-C3-3* was stably identified in two environments (*qHC-13WH-3* and *qHC-14WH-5*), with PVs of 8.57% and 9.20%, respectively. For LC, *cqLC-A10-1* was the sole QTL repeatedly detected in multiple environments (*qLC-13WH-1* and *qLC-15DL-2*), and it exhibited a positive additive effect, indicating that the favorable alleles were derived from the female parent N53-2. In the case of CC, *cqCC-A4-1* was stably detected in two environments (*qCC-15DL-1* and *qCC-14DL-4*), explaining 4.46% and 6.60% of the PV, respectively. A second meta-analysis integrated the 61 consensus QTLs into 58 unique QTLs (Table S6). Three unique QTLs were found to simultaneously regulate two lignocellulose components. For example, *uq.A2-6* and *uq.C1-9* regulated both CC and HC, while *uq.C7-1* affected both LC and HC. These multi-trait QTLs offer valuable insights into the coordinated regulatory mechanisms underlying lignocellulose biosynthesis.

### Identification of candidates within QTL confidence intervals

Based on the syntenic alignment of the high-density genetic linkage map against the *B. napus* reference genome, 4867, 4269, and 7191 candidate genes were identified within the QTL intervals for LC, CC, and HC, respectively. Subsequent Kyoto Encyclopedia of Genes and Genomes (KEGG) pathway enrichment analysis revealed biological pathways associated with each lignocellulose component (Fig. 3B, Fig. S2). Candidate genes linked to LC were predominantly enriched in ‘plant hormone signal transduction’ and ‘phenylpropanoid biosynthesis’, which is consistent with the critical role of phytohormones in activating the transcription of phenylpropanoid pathway genes during lignin biosynthesis. Candidate genes associated with CC and HC were primarily enriched in carbohydrate metabolism pathways, such as ‘galactose metabolism’ and ‘pentose phosphate pathway’. Additionally, 121 structural genes that have been reported to participate in LC, CC, and HC synthesis and regulation in *Arabidopsis thaliana* were compiled (Table S7) [5, 36–39]. Of these, 46 *A. thaliana* genes corresponding to 72 homologous *B. napus* genes were located within the identified QTL intervals, representing strong candidates for lignocellulose regulation.

**Figure 3:**
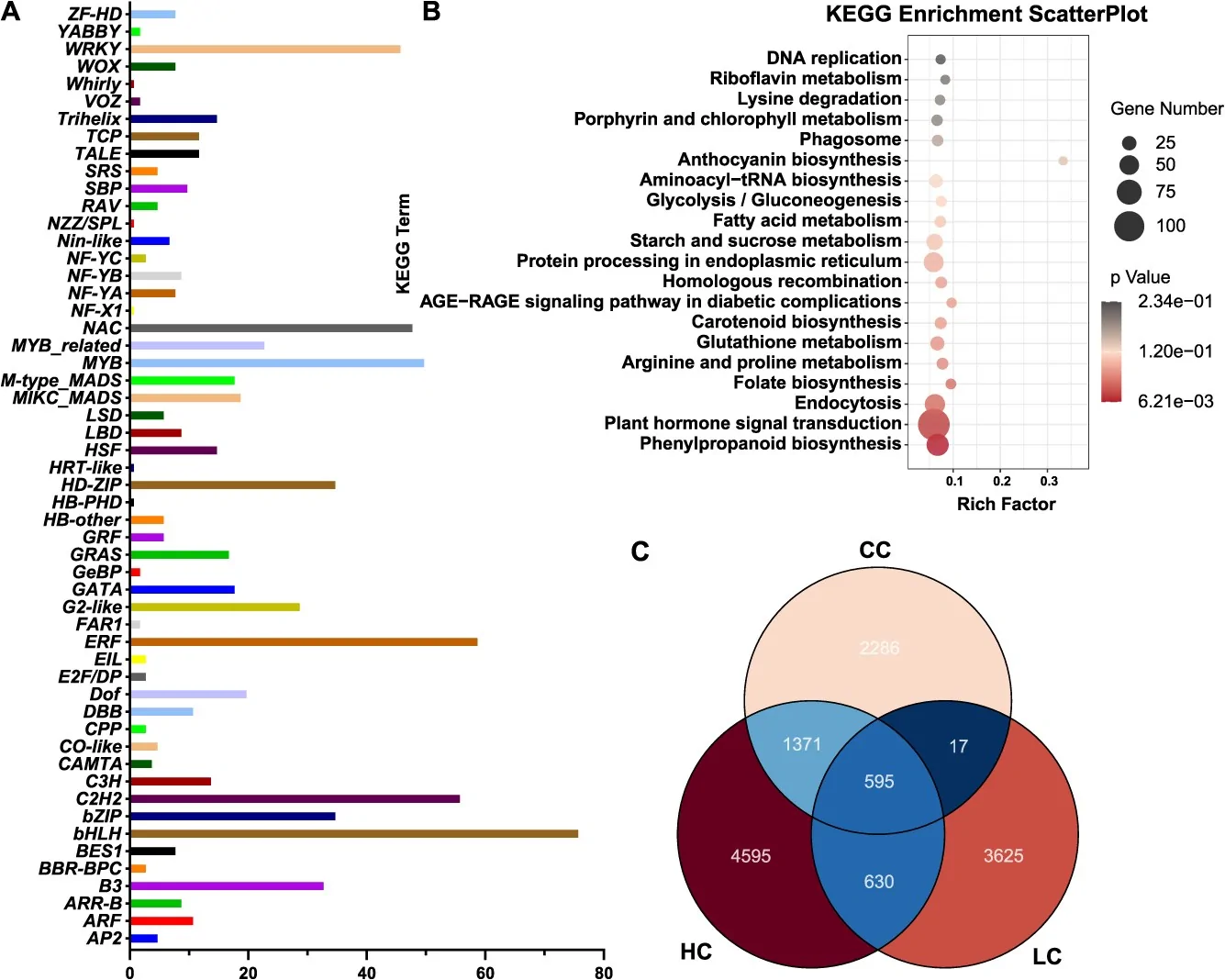
(A) The identified TFs within QTL CIs. The *x*-axis denotes the number of genes for each type of TFs. (B) KEGG pathway enrichment results for LC candidate genes within the CIs (The top 20 most significantly enriched pathways are listed.) The size of each bubble corresponds to the number of enriched genes (gene count), while the shape denotes the statistical significance (*P*-value) of the enrichment. (C) Venn diagram illustrating common candidate genes ascertained within the QTL intervals for LC, CC, and HC.

Gene ontology (GO) enrichment analysis of the 13 119 genes in the QTL confidence intervals (CIs) highlighted ‘sequence-specific DNA binding’, ‘ribonucleoside binding’, and ‘DNA-binding transcription factor activity’ as the most significantly enriched terms (Fig. S3), indicating that lignocellulose biosynthesis is governed by complex transcriptional regulation in *B. napus*. Among the 818 candidate genes annotated as transcription factors (TFs), the most abundant families were bHLH, C2H2, ERF, MYB, WRKY, and NAC (Fig. 3A). Furthermore, a QTL interval on ChrC01 was co-localized for all three lignocellulose components. In this shared region, 595 genes (including 24 TFs) were detected, suggesting potential pleiotropic effects on the coordinated synthesis of lignin, cellulose, and hemicellulose (Fig. 3C, Table S8).

### Integrated analysis of the transcriptome and QTL mapping

To explore the molecular mechanisms regulating the lignocellulose content, stems at the mature stage were selected from high- and low-lignocellulose DH lines for transcriptome analysis. After quality control, adapter removal, and the filtering of low-quality reads, an average of 54.57 million high-quality clean reads per sample, were obtained (Table S9). Over 90% of clean reads were mapped to the *B. napus* reference genome. The reliability of RNA-Seq data was confirmed by quantitative real-time PCR (*q*RT-PCR) analysis of 17 randomly selected genes, which showed a strong concordance with the RNA-Seq expression profiles (Fig. S4). Using the gene expression profile of lines with a low lignocellulose content as the control, differentially expressed genes (DEGs) were identified between the high- and low-lignocellulose lines, enabling a genome-wide analysis of transcriptional differences.

A total of 9199 DEGs (including 4888 up-regulated genes and 4311 down-regulated genes) were detected (Fig. 4A, Table S10). GO enrichment analysis revealed a significant overrepresentation of DNA-binding-related terms, which is consistent with the enrichment of candidate genes in QTL CIs (Fig. S5A). The term ‘cell wall’ was also significantly enriched in cellular component (Fig. S5B). KEGG pathway analysis indicated significant enrichment in ‘phenylpropanoid biosynthesis’, ‘flavonoid biosynthesis’, ‘pentose and glucuronate interconversion’, and ‘starch and sucrose metabolism’ (Fig. 4B).

**Figure 4:**
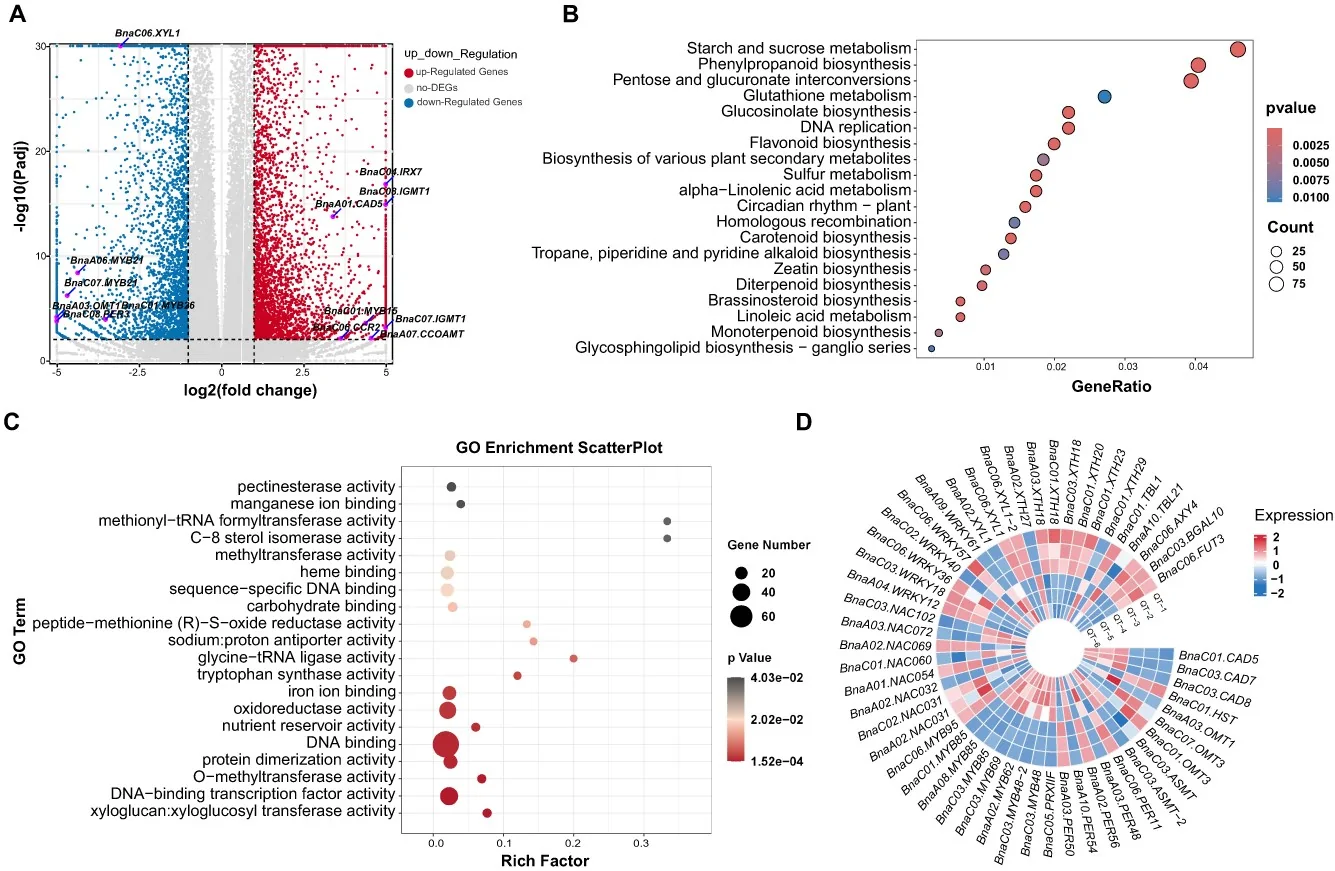
(A) Volcano plot of differential expression analysis of high- and low-lignocellulose DH lines. Each point represents a gene. (B) Significantly enriched KEGG pathways for the 9199 DEGs. Bubble size indicates the number of DEGs per pathway and color intensity corresponds to the statistical significance (*P*-value). Rich factor indicates degree of enrichment (The top 20 most significantly enriched pathways are listed). (C) GO terms enriched among the 1035 common candidate genes (The top 20 most significantly enriched pathways are listed). (D) Expression heatmap of the 52 high-confidence candidate genes. Red and blue shades indicate up- and down-regulation, respectively.

By integrating DEGs with genes located in QTL intervals, 1035 candidate genes (437 and 598 were down-regulated and up-regulated genes, respectively) potentially associated with variation in the lignocellulose content were identified (Fig. S6). These genes were significantly enriched in GO terms related to DNA binding, xyloglucan:xyloglucosyl transferase activity, DNA-binding transcription factor activity and O-methyltransferase activity (Fig. 4C, Table S11). KEGG enrichment analysis revealed their involvement in ‘flavonoid biosynthesis’, ‘galactose metabolism’, ‘starch and sucrose metabolism’, ‘phenylpropanold biosynthesis’, and ‘Pentose and glucuronate interconversions’ (Fig. S7). The ‘plant hormone signal transduction’ pathway was also enriched, indicating the potential role of phytohormones in regulating lignocellulose biosynthesis in rapeseed stems. Among the 72 *B. napus* genes homologous to *A. thaliana* structural genes in the QTL CIs, 30 exhibited significantly differential expression between the high- and low-lignocellulose lines (Table S12), highlighting them as strong candidate genes for stem lignocellulose regulation.

### Regulatory network construction and key TF identification

Co-expression analysis of 1035 candidate genes was conducted to elucidate the regulatory mechanism underlying the stem lignocellulose content (Fig. S8). Genes involved in TF regulation, starch and sucrose metabolism, phenylpropanoid biosynthesis, biosynthesis of amino acids, and plant hormone signal transduction were selected to build the regulatory network. Network topology analysis, particularly the node degree, identified several TFs as key regulators with multiple downstream targets (Fig. 5). Among them, *BnaC06.ARF8*, *BnaC05.RGL2*, *BnaA01.NAC054*, and *BnaA05.RGL2* exhibited the highest connectivity, suggesting that they serve as central hubs in lignocellulose biosynthesis by modulating multiple pathways. Protein–protein interaction predictions indicated that these TFs likely interact, potentially forming a functional complex. Critical structural genes, including *BnaC06.PER11*, *BnaA03.PER50*, *BnaA03.PER48*, *BnaA03.OMT1*, *BnaC07.OMT3*, *BnaC03.CAD8*, *BnaC01.HST*, *BnaA02.XYL1*, and *BnaC01.TBL1*, were identified as downstream targets.

**Figure 5:**
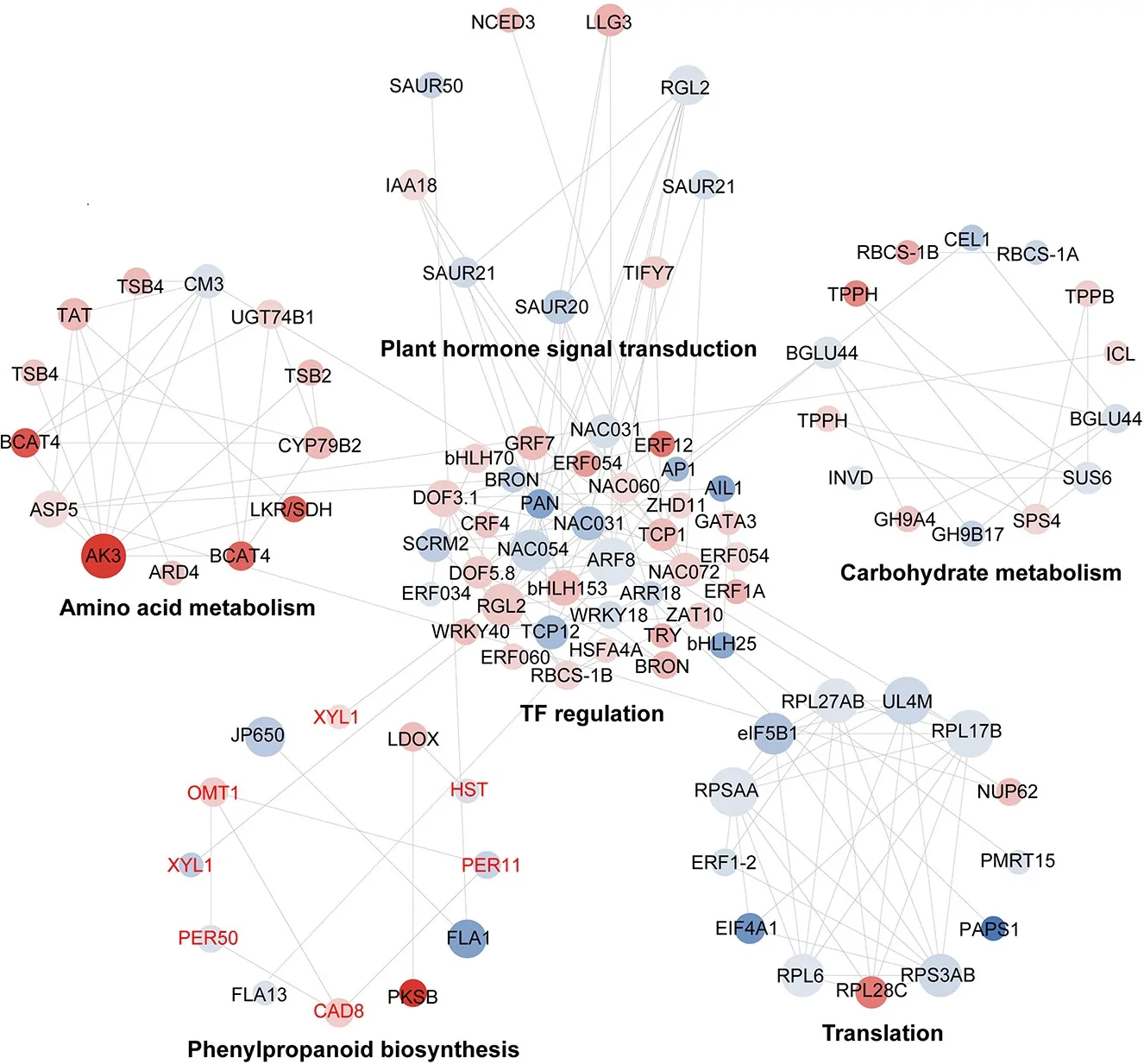
The network of TFs and related pathways. Colors represent the expression level of candidate genes in high- and low-lignocellulose lines. The size of the circle represents the number of nodes.

R2R3-MYB, NAC, and WRKY TFs are well established as regulators of lignin biosynthesis and secondary cell wall formation [40]. In this study, 50 *MYB*, 48 *NAC*, and 46 *WRKY* genes were located within QTL intervals. By integrating DEGs, 22 TFs (8 *MYB*, 8 *NAC*, and 6 *WRKY* genes) were retained as high-confidence candidate genes (Table S13, Fig. S9). Key examples include *MYB85* homologs (*BnaC03.MYB85*, *BnaA08.MYB85*, and *BnaC01.MYB85*) and *WRKY12* (*BnaA04.WRKY12*), suggesting their potential regulatory roles in lignocellulose biosynthesis [41, 42].

### Lignocellulose biosynthetic pathway in *B. napus*

The expressions profiles of 52 high-confidence candidate genes associated with lignocellulose biosynthesis were analyzed in high- and low-lignocellulose DH lines (Figs 4D and 6). Significant differences were observed in genes encoding enzymes and TFs involved in lignocellulose biosynthesis. *BnaC01.HST* was downregulated in high-lignocellulose lines, while *BnaA03.OMT1* and all CAD genes (*BnaC01.CAD5*, *BnaC03.CAD7*, and *BnaC03.CAD8*) were upregulated. The peroxidases *BnaA10.PER54* and *BnaC05.PRXIIF* were upregulated, whereas most hemicellulose-related glycosyltransferase (*GT*) genes—except for *BnaA02.XYL1*, *BnaA02.XTH27*, *BnaC01.XTH29*, and *BnaA10.TBL21*—were downregulated in high-lignocellulose lines.

**Figure 6:**
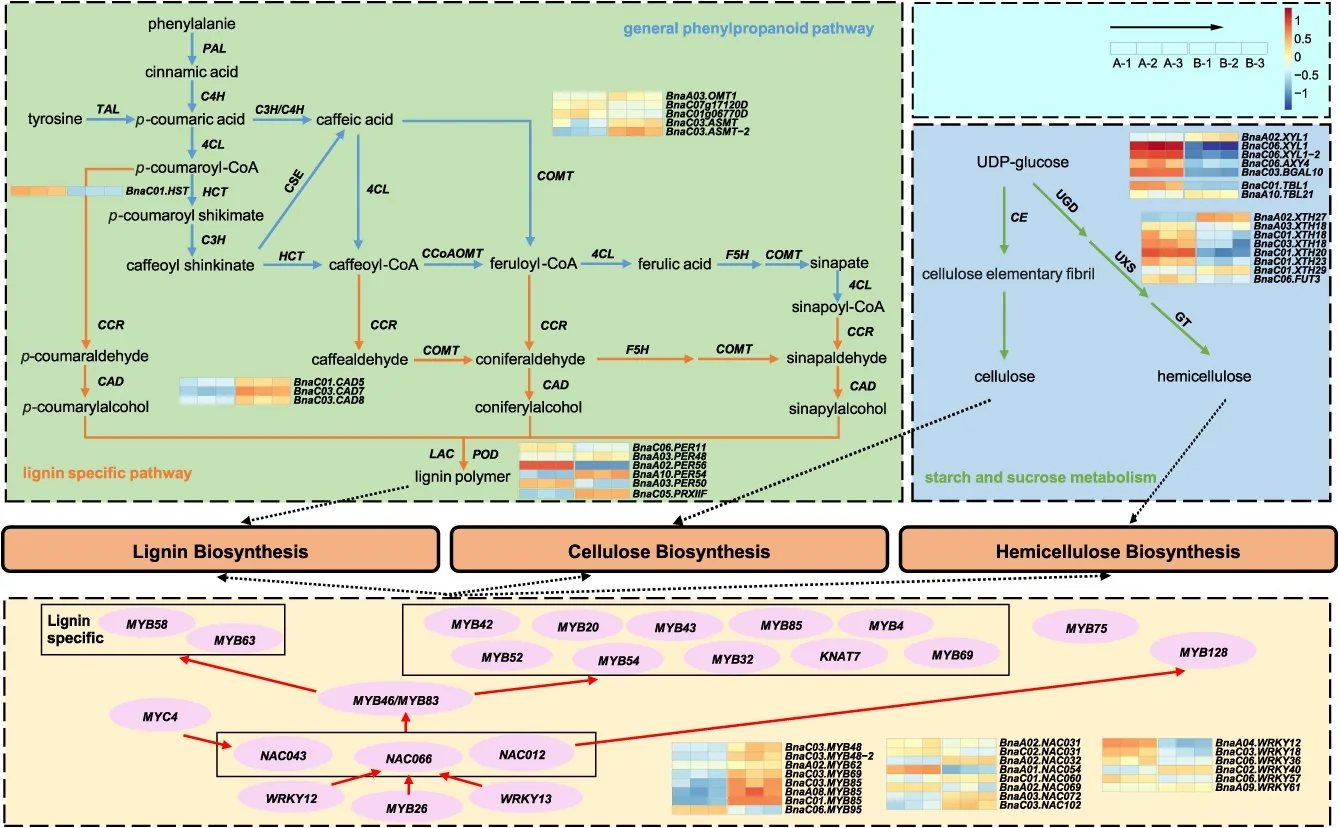
Lignocellulose biosynthesis model in stem. The samples from left to right represent A-1, A-2, A-3, B-1, B-2 and B-3. A-1 ~3 represent low-lignocellulose lines, while B-1 ~3 represent high-lignocellulose lines. Gene expression levels are represented by log_2_(FPKM) values averaged across replicates, as indicated by the color bar: orange and blue colors signify high and low expression, respectively. Red triangles highlight genes that were differentially expressed between the high- and low-lignocellulose lines.

Several TFs exhibited notable differential expression. *BnaA01.NAC054* and *BnaA04.WRKY12* were downregulated in high-lignocellulose lines, suggesting their roles as negative regulators, whereas *MYB85* homologs (*BnaC03.MYB85*, *BnaA08.MYB85*, and *BnaC01.MYB85*) were upregulated, consistent with their positive regulatory roles in lignin biosynthesis. Tissue-specific expression analysis revealed that *BnaC01.MYB85*, *BnaA04.WRKY12*, and *BnaC03.ASMT* were preferentially expressed in stems (Fig. S10), supporting their functions in stem lignocellulose biosynthesis.

### Haplotype analysis of candidate genes

Considering their preferential expression in stem tissue, *BnaC01.MYB85, BnaA04.WRKY12*, and *BnaC03.ASMT* were selected for haplotype analysis. A natural population comprising 400 lines was collected to investigate the distribution patterns of *BnaC01.MYB85*, *BnaA04.WRKY12*, and *BnaC03.ASMT* in *B. napus*. For haplotype analysis, full-length sequences of the three genes were amplified across all lines. Although *BnaC01.MYB85* and *BnaA04.WRKY12* did not exhibit distinct haplotypes in this population (Figs S11 and S12), *BnaC03.ASMT* showed two major haplotypes (frequency > 5%), namely as hap01 (*n* = 178) and hap02 (*n* = 53) (Fig. 7A and B). Two nonsynonymous SNPs were detected in the exon regions of *BnaC03.ASMT*, resulting in the amino acid substitutions of asparagine at position 154 with lysine and threonine at position 381 with methionine. The substitution at position 154 was predicted to disrupt a key functional domain (pfamA ID: Methyltransf_2), potentially impairing protein function (Fig. S13). The tertiary structures of the hap01 and hap02 protein variants of *BnaC03.ASMT* were predicted using AlphaFold3, and the highest confidence model was selected for each. Subsequent visualization and comparative analysis in PyMOL revealed structural differences between the two haplotypes (Fig. 7D–G). Phenotypic analysis indicated that lines carrying hap01 exhibited a significantly higher HC than those carrying hap02 in the natural population (Fig. 7C), suggesting a functional link between the observed structural variation and the lignocellulose phenotype.

**Figure 7:**
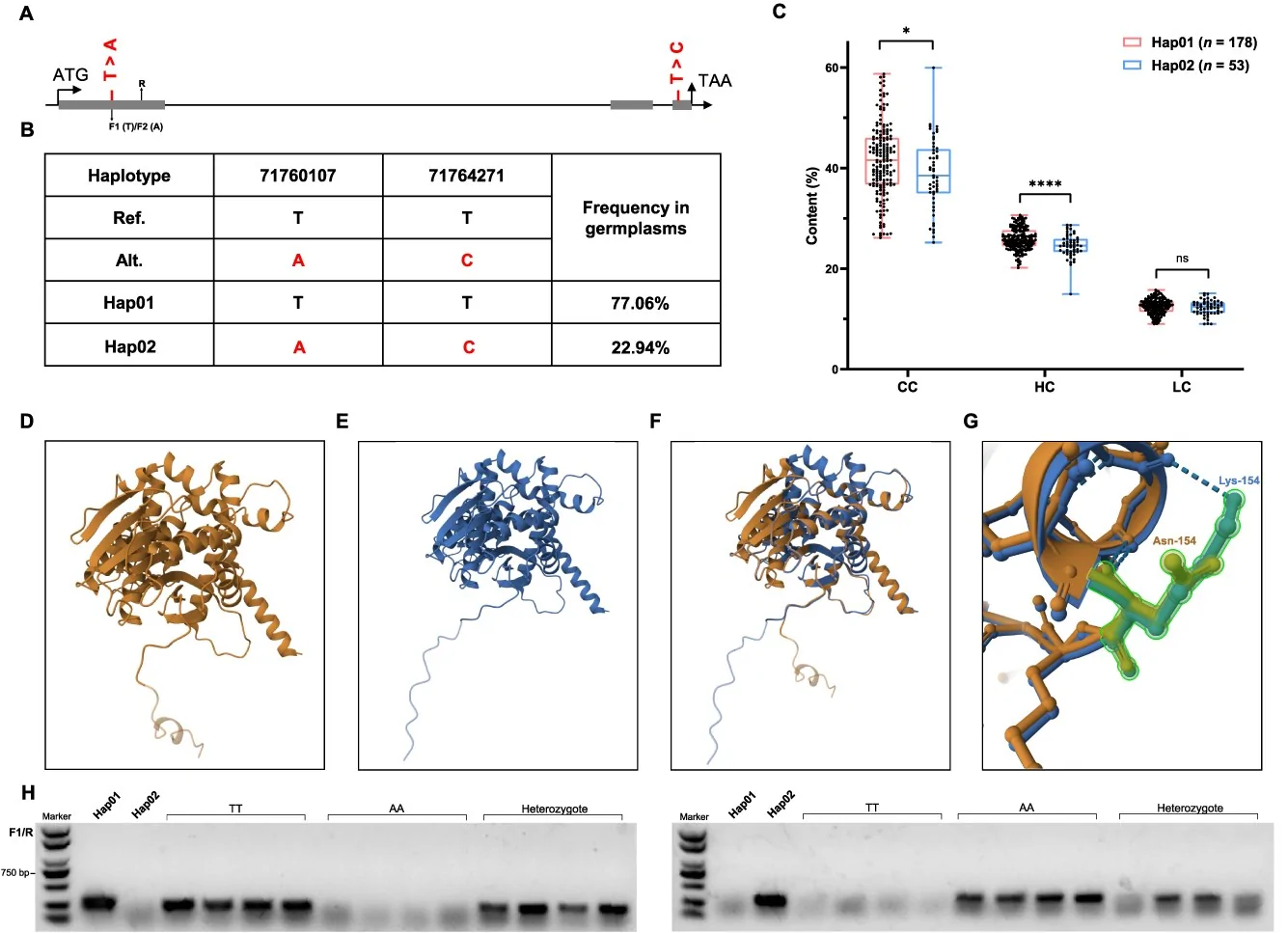
Haplotype analysis of *BnaC03.ASMT*. (A and B) Sequence alignment of the two major haplotypes (hap01 and hap02) of *BnaC03.ASMT* identified in a natural population of *B. napus*. The reference sequence (Ref.) derived from cultivar ZS11 is included for comparison. Polymorphic sites are explicitly indicated. (C) LC, CC, and HC in lines carrying hap01 or hap02. Statistical significance is indicated as follows: ^*^, *P* < 0.05; ^****^, *P* < 0.0001; ns, not significant (*P* > 0.05). (D and E) Predicted tertiary structures of the hap01-type (D) and hap02-type (E) proteins. (F) Superimposed view of the tertiary structures of the hap01 and hap02 types. (G) Key binding sites where amino acid variations affect the protein’s tertiary structure. (H) Confirmation of co-segregated makers (F1/R; F2/R) in the natural population.

Haplotype analysis was also conducted for the remaining high-confidence candidate genes. Of these, 15 candidates formed distinct haplotypes, 4 of which showed significant phenotypic differences among haplotypes (Tables S14–S28, Figs S14 and S15). Another copy of ASMT on ChrC03 (*BnaC03.ASMT-2*) also exhibited significant PV among its haplotypes (Fig. S15), emphasizing its role in regulating the lignocellulose content.

Allele-specific PCR markers based on sequence polymorphisms between hap01 and hap02 were developed to facilitate the rapid identification of *B. napus* individuals carrying distinct *BnaC03.ASMT* haplotypes. Two primer pairs, ASMT.F1/ASMT.R and ASMT.F2/ASMT.R, were designed to serve as co-dominant markers (Fig. 7H; Table S29). PCR amplification with the ASMT.F1/ASMT.R primer set yielded a 282-bp fragment specific to hap01 (present in hap01 homozygotes and heterozygotes), whereas no product was amplified from hap02 homozygotes (Fig. 7H). The ASMT.F2/ASMT.R primer amplified the same-sized fragment in hap02 homozygotes and heterozygotes, but not in hap01 homozygotes. These markers, which were verified in the DH population (Fig. S16), enabled the clear and efficient discrimination of the two haplotypes for breeding applications.

## Discussion

*Brassica napus* is an important crop worldwide, providing vegetable oil, animal feed, and biofuel [1]. Lodging significantly reduces the mechanized harvesting efficiency and leads to yield losses in *B. napus* [2, 3]. Stem strength, which is largely determined by the lignocellulose content, is a key determinant of lodging resistance [43]. Thus, elucidating the genetic and regulatory mechanisms controlling the stem lignocellulose content is crucial for improving yield, enabling lodging resistance, and supporting large-scale mechanized cultivation in rapeseed production.

In this study, we measured the contents of three lignocelluloses in stems across six environments, and we observed substantial variation with an approximately normal distribution, indicating that these traits are quantitatively inherited. The three lignocellulose components were positively correlated with each other and with multiple yield-related traits, highlighting the agronomic significance of stem lignocellulose. However, breeding rapeseed for dual purposes—oil production and vegetable use—results in a significant trade-off; although lignocellulose accumulation is fundamental for achieving high biomass and lodging resistance, it detrimentally affects sensory attributes such as tenderness and crispness. Consequently, fine-tuning lignocellulose biosynthesis and deposition is a pivotal strategy for developing rapeseed cultivars that harmonize agronomic productivity with desirable eating quality.

QTL mapping (including linkage mapping and GWAS) has been widely used in quantitative trait analysis for the agronomic characteristics of *B. napus* [31–33]. Genome-wide QTL mapping identified 65 initial QTLs for stem lignocellulose traits across six microenvironments, which were consolidated into 61 consensus QTLs using meta-analysis. Among these, four QTLs were environmentally stable, and three unique loci showed pleiotropic effects, regulating multiple lignocellulose components. These robust QTLs represent promising targets for marker-assisted selection. QTLs for stem lignocellulose were predominantly located on ChrA06 and ChrC01, contrasting with seed fiber QTLs, which have been reported on ChrA09 [23, 27], suggesting that stems and seeds are regulated by different genetic loci. Moreover, candidate genes, such as *BnaC03.CAD7*, *BnaC01.XTH20* and *BnaC03.BGAL10*, which are homologous to the *A. thaliana* lignocellulose biosynthesis genes, were identified based on QTL mapping of the KN DH population. These results confirm the accuracy of our QTL mapping, and these novel QTLs may be important genes for anti-lodging traits in rapeseed.

Integrative multi-omics analysis has frequently been used to narrow down the candidate genes within QTL intervals, significantly enhancing the reliability and reproducibility of candidate gene identification. In this study, an integrative analysis was performed on the DEGs obtained from the comparative transcriptome analysis of high- and low-lignocellulose DH lines and the candidate genes within the QTL intervals. This analysis yielded 1035 candidate genes, among which 30 high-confidence candidate genes in QTL intervals exhibited significant differential expression. Key structural genes, such as *BnaA03.OMT1*, which encodes caffeic acid/5-hydroxyferulic acid O-methyltransferase—critical for the synthesis of syringyl lignin—were upregulated in high-lignocellulose lines [44].

In addition to these key enzymes, many TFs were also identified. WRKY, NAC, and MYB TFs have been reported to regulate cell wall biosynthesis and lignin synthesis, but only a few have been demonstrated to directly regulate lignin biosynthesis genes [40, 45]. These TFs bind to the promoters of downstream target genes and interact with other regulators, having activating or repressive effects on lignin, cellulose, and hemicellulose biosynthesis. In this study, eight MYB, eight NAC, and six WRKY genes were identified, some of which are homologous to *A. thaliana* genes, such as *BnaA08.MYB85*, *BnaC01.MYB85*, and *BnaA04.WRKY12*, with established roles in lignin, cellulose, and hemicellulose biosynthesis [40–42, 46]. Building on our previously established TF interaction network in *B. napus* seed [47], *BnaA02.NAC032*, which was identified in this study, was found to interact with *BnaC06.MYB63*. Given that the *A. thaliana* orthologous *AtMYB63* activates the lignin biosynthetic genes and causes growth retardation when overexpressed [48], the expression of *BnaC06.MYB63* was significantly upregulated in the stems of high-lignocellulose lines, suggesting a relatively conserved regulatory module in seeds and stems. Although *BnaC06.MYB63* integrates signals from multiple regulators [47], *BnaA02.NAC032* may modulate the spatial expression dynamics of *BnaC06.MYB63* through protein interaction and further regulate lignin biosynthesis.

Haplotype analysis has great potential for the identification and deployment of superior haplotypes in plant breeding. Breeders can classify germplasm into groups based on haplotype combinations associated with desirable phenotypes. This ‘haplotype-based breeding’ strategy enables the direct selection of optimal allele combinations, effectively pyramiding favorable alleles at multiple loci within a haplotype block to develop improved varieties. In this study, *BnaC03.ASMT* was divided into two major haplotypes, with hap01 associated with a higher HC than hap02. Structural modeling predicted that a nonsynonymous substitution in hap02 would disrupt a key functional domain, potentially impairing enzyme function. The identified superior haplotype and its carrier accessions provide valuable resources for developing improved lodging-resistant rapeseed cultivars through haplotype-based breeding. The development of allele-specific PCR markers enables the rapid selection of favorable haplotypes for breeding. Although ASMT is a recognized key enzyme in melatonin production, its potential function in lignocellulose synthesis remains unknown. In Gossypium species, *GhOMT33_Dt* (a homolog of *ASMT*) exhibits differential expression during fiber development [49].

In summary, our integrative analysis combining QTL mapping, transcriptomics, and haplotype profiling delineated the genetic architecture of stem lignocellulose in *B. napus*. To provide more information to illuminate the genetic regulation mechanism of lignocellulose, we integrated 30 high-confidence structural genes and 22 TFs from the DEGs and QTL CIs to construct a specific pathway for lignocellulose metabolism in *B. napus* stems. The differential expression of these key enzymes and TFs might affect lignocellulose synthesis, thereby changing the stem hardness. Several candidate genes were identified in this study, but their precise functions require experimental validation. These findings provide not only insights into the molecular regulation of lignocellulose biosynthesis but also valuable resources for the molecular breeding of lodging-resistant rapeseed cultivars.

## Materials and methods

### Plant materials and growth conditions

A 300-line DH population (named KN DH) developed from a cross between female parent N53-2 and male parent Ken-C8 [50] was used to detect QTLs related to stem lignocellulose components. The KN DH population and its parental lines were cultivated over three consecutive growing seasons from 2012 to 2015 at two contrasting locations in China: WH (Hubei Province; semi-winter rapeseed region, October–May) and DL (Shaanxi Province; winter-type rapeseed region, September–June). Plants were arranged with a row spacing of 40 cm and an in-row plant spacing of 20 cm. Standard agronomic management practices were applied at both sites.

### Lignocellulose content analysis and generalized heritability estimation

Stem samples were harvested from five randomly selected plants per DH line at 20 cm above the cotyledon scar. Samples were dried, ground into a fine powder, and subjected to near-infrared reflectance spectroscopy (NIRS) on a FOSS NIRS 5000 instrument with WinISI software, following established protocols [25]. The phenotypic values for acid detergent lignin, acid detergent fiber, and neutral detergent fiber were derived from NIRS spectra using pre-calibrated models [25], and LC, CC, and HC were calculated as previously described [24].

Broad-sense heritability (*H^2^*) was estimated with IBM SPSS Statistics 25 (IBM Corporation, USA) using the following formula: *H^2^* = *V_G_* / *V_P_* = *V_G_* / (*V_G_* + *V_e_* / *r*), where *V_G_*, *V_p_*, and *V_e_* represent the genotype variance, total phenotypic variance, and genotype-by-environment interaction variance, respectively, and *r* denotes the number of survey periods within each environment.

### QTL mapping and integration of lignocellulose components

The KN DH population and a high-density SNP-based linkage map [50] were used for QTL analysis. Composite interval mapping (CIM) was performed using Windows QTL Cartographer 2.5 (http://statgen.ncsu.edu/qtlcart/WQTLCart.htm), with the following parameters: a walking speed of 2 cM, a window size of 10 cM, and five background cofactors. The logarithm of odds (LOD) threshold for significant QTLs was determined via permutation tests (1000 repetitions) at a 5% experiment-wise error rate.

QTLs in the same genomic region were integrated into consensus QTLs for each trait through meta-analysis based on environmental factors using BioMercator 2.1 [51], and these consensus QTLs were consolidated into unique QTLs through a second meta-analysis based on traits. A designation using ‘*q*’, ‘*cq*’, or ‘*uq*’, followed by the trait abbreviation (LC, CC, or HC), the linkage group (A01–A10 or C01–C09), and a sequential number, were applied to name the identified, consensus, and unique QTLs, respectively, following a previous description [52]. CIs were defined by 2-LOD support intervals. Candidate genes within the QTL CIs were identified as previously described [39] and annotated based on *A. thaliana* orthologues (TAIR: https://www.arabidopsis.org/index.jsp).

### RNA sequencing and differential expression analysis

Total RNA was extracted from high- and low-lignocellulose lines, and cDNA libraries were constructed and sequenced as previously reported [53]. High-throughput sequencing was performed on the DNBSEQ-T7 platform to generate raw reads (150 bp, paired-end). The raw sequencing data are available in the NCBI Sequence Read Archive (SRA, https://www.ncbi.nlm.nih.gov/sra) under accession PRJNA1342351.

Raw reads were processed using fastp [54] to remove reads with an *N* content exceeding 10% and those with more than 50% low-quality bases (*Q* ≤ 5). Clean reads were aligned to the Darmor-*bzh* (v4.0) reference genome using HISAT2 [55], and only uniquely mapped reads were retained for further analysis. Gene expression levels were quantified using StringTie and Ballgown [56], with genes showing a cumulative read count exceeding 10 across three biological replicates considered as expressed.

Sample correlation heatmaps were generated using pheatmap (https://github.com/raivokolde/pheatmap). DEG analysis was performed with DESeq2 [57] using thresholds of false discovery rate (FDR) ≤ 0.01 and |log_2_(fold change)| ≥ 2. Venn diagrams were created using TBtools v1.076 [58], and UpSet diagrams were produced with OmicShare tools (http://www.omicshare.com/tools).

Functional annotation of Darmor-*bzh* genes was performed using Blast2GO [59] and InterProScan 5 [60]. GO and KEGG enrichment analyses were conducted using TBtools v1.076, with significant terms identified at a Benjamini–Hochberg corrected *P*-value (*q*-value) <0.05. Representative GO terms were extracted using REVIGO (http://revigo.irb.hr/) to eliminate redundancy. Expression heatmaps were visualized using TBtools v1.076.

### Gene regulatory network construction

Potential interaction networks for candidates were retrieved from the STRING database (http://string-db.org) and imported into Cytoscape software (version 3.9.0) [61] for visualization. Node degrees, which represent the number of connections with other nodes, were calculated to identify the central regulators in Cytoscape.

### qRT-PCR validation of RNA-Seq data

Seventeen DEGs were randomly selected for validation. cDNA was synthesized from the same RNA samples used for RNA-Seq using a RevertAid First Strand cDNA Synthesis Kit (Thermo Fisher Scientific, USA) following the standard operating procedure provided by the manufacturer. Specific primers were designed based on multiple sequence alignment (Table S1), with *ACT7* as an internal control. *q*RT-PCR was performed using ChamQ Universal SYBR® *q*PCR Master Mix (Vazyme Biotech Co. Ltd., Nanjing, China) on a Bio-Rad CFX384™ Real-Time System (Bio-Rad, Hercules, CA, USA). There were three biological replicates and three technical replicates per sample. Relative expression was calculated using the 2^(−ΔΔCT) method [62].

### Haplotype analysis and protein tertiary structure prediction

Full-length sequences of 52 high-confidence candidates were amplified from a natural population comprising 400 *B. napus* lines for haplotype classification. Significant differences in LC, CC, and HC were determined among haplotypes. Amino acid sequences for the different haplotypes were translated using the NovoPro online tool (https://www.novopro.cn/tools/translate.html). Protein tertiary structures were predicted using AlphaFold3 (https://alphafoldserver.com/) and visualized in PyMOL (v3.1.5.1).

## Supplementary Material

Web_Material_uhag201

## Data Availability

All relevant data are available within this article and supplemental information. Gene and protein sequences are publicly accessible in TAIR (https://www.arabidopsis.org/) and BnIR (https://yanglab.hzau.edu.cn/BnIR).
