# Supplementary material for: Integrative QTL mapping and transcriptomic profiling uncover molecular mechanisms underlying lodging resistance in Brassica napus
Source: Hortic Res. 2026 May 20;13(10):uhag201. doi: 10.1093/hr/uhag201 (PMC13633099; doi:10.1093/hr/uhag201)
Supplement: Supplementary file 1 [file web_material_uhag201.zip › Supplementary Figures.docx]

**Supplementary Figures**

**Figure S1.** The correlational analyses of (**A**) CC; (**B**) HC; (**C**) LC in different environments.

**Figure S2.** KEGG enrichment analyses were performed for all genes potentially regulated CC (**A**) and HC (**B**) within QTL CIs. The bubble size and shape respectively denote the gene count and *p*-value associated with each enriched term.

**Figure S3.** GO enrichment analyses were performed for all genes potentially regulated three lignocellulose contents within QTL CIs. The bubble size and shape respectively denote the gene count and *p*-value associated with each enriched term.

**Figure S4.** Confirmation of transcript abundance via RT-qPCR. **A** and **B** represent low- and high- lignocellulose lines, respectively.

**Figure S5**. The GO enrichment analysis of 9,199 DEGs (**A**) and the network based on the Top5 GO terms in Cellular Component (**B**).

**Figure S6**. Venn diagram illustrating common genes through QTL and DEGs.

**Figure S7**. Bubble plot of KEGG enrichment analysis for 1,035 candidate genes: bubble dimensions reflect gene counts per term, while bubble shapes indicate their statistical significance (*p*-values).

**Figure S8**. The network of DEGs and related pathways of phenylpropanoid, starch and sucrose, plant hormone, amino acids, and TF regulations. Colors represent the expression level of candidate genes in high- and low-lignocellulose lines. The size of the circle represents the number of nodes.

**Figure S9.** The expression level of 22 candidate TFs and structural genes within the identified QTL intervals that reported to participate in the synthesis and regulation of LC, CC and HC.

**Figure S10.** The expression level of *BnaC01.MYB85*, *BnaA04.WRKY12* and *BnaC03.ASMT* in different tissues.

**Figure S11.** Haplotype analysis of *BnaC01.MYB85*.

**Figure S12.** Haplotype analysis of *BnaA04.WRKY12*.

**Figure S13.** Sequence alignment of the two major haplotypes (hap01 and hap02) of *BnaC03.ASMT* identified in a natural population of *B. napus*.

**Figure S14**. CC, HC and LC among rapeseed accessions with different haplotypes of *BnaA01.NAC054*, *BnaC06.XYL1* and *BnaA02.XTH27*. LC, Lignin content; CC, Cellulose content; HC, Hemicellulose content. Statistical significance is indicated as follows: *, *p* < 0.05; ***, *p* < 0.001; ns, not significant (*p* > 0.05).

**Figure S15**. Haplotype analysis of *BnaC03.ASMT-2*. (**A**) Three major haplotypes (Hap01, Hap02 and Hap03) of *BnaC03.ASMT-2* identified in a natural population of *B. napus*. Polymorphic sites are explicitly indicated. (**B-D**) CC, HC and LC in lines carrying Hap01, Hap02 or Hap03. LC, Lignin content; CC, Cellulose content; HC, Hemicellulose content. Statistical significance is indicated as follows: *, *p* < 0.05; ***, *p* < 0.001; ns, not significant (*p* > 0.05).

**Figure S16**. Confirmation of co-segregated makers (F1/R; F2/R) in the KN DH population.
